# Validation of the midface volume deficit scale using a new photo‐guideline in the Asian population

**DOI:** 10.1111/dth.12938

**Published:** 2019-04-29

**Authors:** Guk Jin Jeong, Hye Sung Han, Ga Ram Ahn, Eun Jung Ko, Sun Young Choi, Keyong Ho Lee, Beom Joon Kim

**Affiliations:** ^1^ Department of Dermatology Chung‐Ang University College of Medicine Seoul Republic of Korea; ^2^ Department of Dermatology National Police Hospital Seoul Republic of Korea; ^3^ Department of Dermatology Seoul Paik Hospital, Inje University College of Medicine Seoul Republic of Korea; ^4^ Department of R&D, Across Co., Ltd. Chuncheon Republic of Korea

1

With the development of cosmetic treatment options for age‐related midface changes, such as volume loss, there is an increasing need for validated scales to objectively evaluate clinical outcomes and facilitate the optimal use of products for cosmetic facial treatment (Jones & Murphy, 2013). The midface volume deficit scale (MFVDS) is an Allergan®‐specific scale that uses a six‐point photo‐numeric instrument specifically developed as a physician's assessment tool to evaluate the overall degree of the midface volume deficit, with grades ranging from 0 (none) to 6 (severe). A description of each grade in the MFVDS is shown in Table 1 (Urdiales‐Galvez et al., 2017). The structural and anatomical features specific to the Asian face and the characteristics of facial aging that differ from those in Western populations necessitate validation of the MFVDS in an Asian population (Vashi, de Castro Maymone, & Kundu, 2016). Thus, we performed a validation study of the MFVDS in an Asian population using a photo‐guideline that was created using the grading system of the MFVDS with the corresponding photographs of our Asian subjects.

**Table 1:** Allergan® midface volume deficit scale

Grade	Definition
0 None	Moon face *Fullness* (*convexity*) in the zygomaticomalar region, anteromedial cheek, and/or submalar region
1 Minimal	*Flattening* in the zygomaticomalar region, anteromedial cheek, and/or submalar region
2 Mild	*Mild concavity* in the zygomaticomalar region, anteromedial cheek, and/or submalar region Mild *tear troughs and/or nasolabial folds*
3 Moderate	*Moderate concavity* in the zygomaticomalar region, anteromedial cheek, and/or submalar region Moderate tear troughs and/or nasolabial folds Mild *nasojugal folds and/or prejowl sulcus* Mild prominence of bony landmarks Mild visibility of musculature
4 Significant	*Significant concavity* in the zygomaticomalar region, anteromedial cheek, and/or submalar region Significant tear troughs and/or nasolabial folds Moderate nasojugal folds and/or prejowl sulcus Moderate prominence of bony landmarks Moderate visibility of musculature
5 Severe	*Wasting* Severe concavity in the zygomaticomalar region, anteromedial cheek, and/or submalar region Severe tear troughs and/or nasolabial folds Significant nasojugal folds and/or prejowl sulcus Significant prominence of bony landmarks Significant visibility of underlying musculature

The validation study included 38 Asian subjects whose photographs were assessed independently by six experienced board‐certified dermatologists. During the evaluation, a photograph was given an MFVDS grade when four of six evaluators reached a consensus. When five of six evaluators reached a consensus, the photograph was selected as a candidate for the photo‐guideline. Among the selected candidate photographs, one photograph of each grade was finally selected as the scale photograph. Using the six selected scale photographs, the photo‐guideline was created (Figure 1). For the validation assessment of the MFVDS, seven less‐experienced clinicians were trained with the photo‐guideline. After training, the clinicians evaluated the remaining 32 set photographs that were not used in the photo‐guideline. During the evaluation, 14 set photographs were repeated in order to assess the intra‐rater reliability. As a result, each clinician evaluated a total of 46 set photographs for severity of volume loss or midface contour deficiency and scored each photograph using MFVDS grades. An overall weighted kappa coefficient and corresponding 95% confidence interval were used to assess the inter‐ and intra‐rater reliabilities. For both within‐ and between‐observer agreement, weighted kappa coefficients were interpreted as follows: 0–0.19, poor agreement; 0.20–0.39, fair agreement; 0.40–0.59, moderate agreement; 0.60–0.79, substantial agreement; and 0.80–1.0, almost‐perfect agreement (Landis & Koch, 1977). The intra‐rater weighted kappa coefficients (Cohen kappa) for agreement in the MFVDS grades ranged from 0.7627 to 1.0000, indicating substantial to almost‐perfect agreement to produce identical MFVDS grades for the same subject (Table 2). The overall weighted kappa coefficient (Fleiss kappa) for inter‐rater agreement was 0.63339, indicating substantial agreement (Table 2). On the basis of the interpretation of the weighted kappa coefficients, high degrees of intra‐ and inter‐rater agreement were observed when using the MFVDS in Asians.

**Figure 1:**
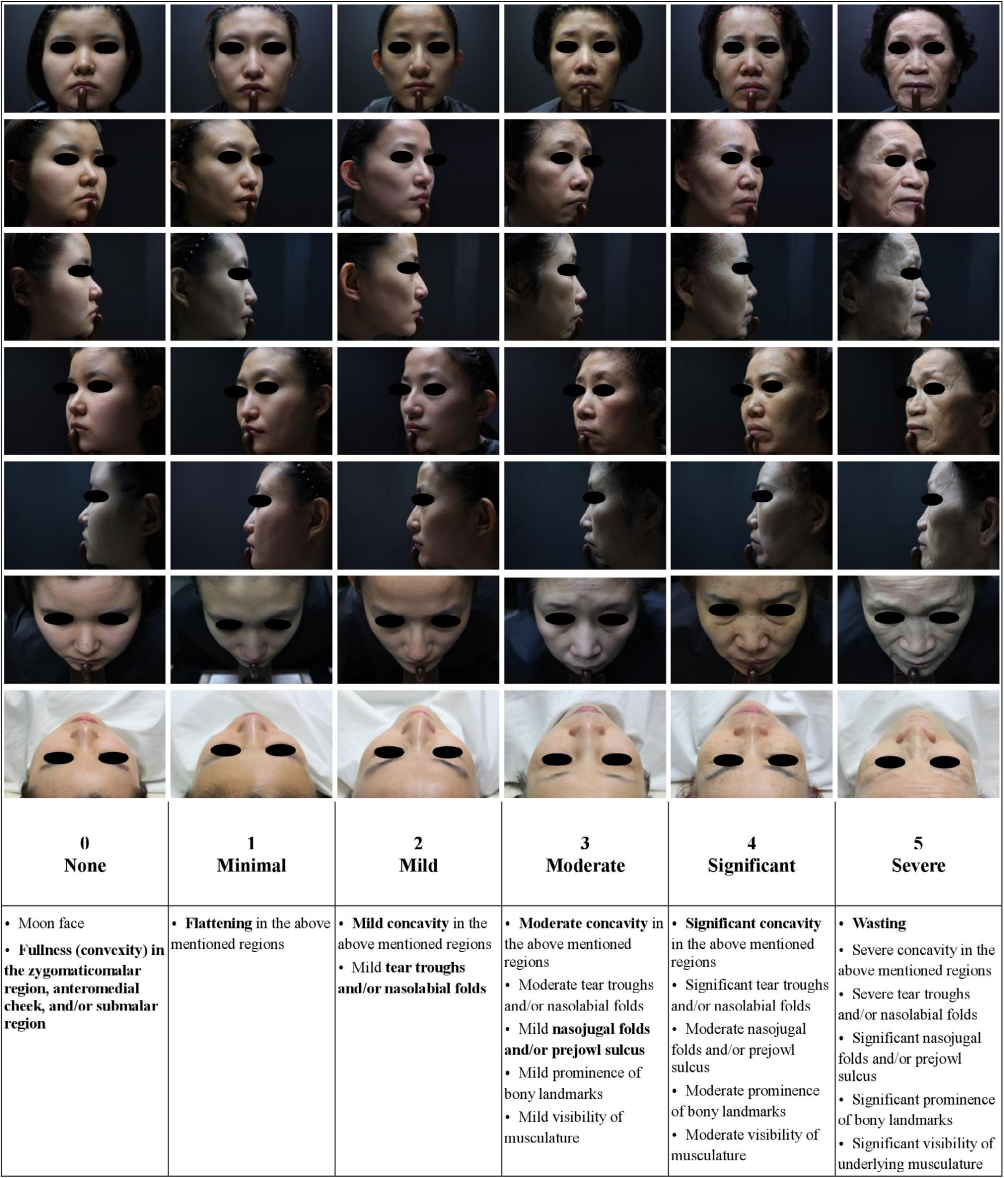
Photo‐guideline of the midface volume deficit scale in the Asian population

**Table 2:** Intra‐rater weighted kappa coefficients (Cohen kappa) and overall inter‐rater weighted kappa coefficients (Fleiss kappa) of the midface volume deficit scale in the Asian population

Investigator	Cohen kappa	95% CI
1	0.8205	(0.6834, 0.9576)
2	1.0000	(1.0000, 1.0000)
3	0.7627	(0.6094, 0.9160)
4	0.9200	(0.8121, 1.0000)
5	1.0000	(1.0000, 1.0000)
6	0.9200	(0.8096, 1.0000)
7	1.0000	(1.0000, 1.0000)
	*Fleiss kappa*	*95% CI*
Overall	0.63339	(0.60837–0.65840)

To our knowledge, this is the first validation study on the use of the MFVDS in the Asian population. The high degree of intra‐ and inter‐evaluator agreement suggests that the MFVDS can be used by different investigators on different occasions and by the same investigator on different occasions to reliably assess midface volume or the change in volume after treatment. These results support the suitability of the MFVDS for use in clinical trials targeting Asian subjects to objectively grade midface volume.

## CONFLICT OF INTEREST

The authors declare no conflict of interest.

## FUNDING INFORMATION

This research was supported by a grant of the Korea Health Technology R&D Project through the Korea Health Industry Development Institute (KHIDI), funded by the Ministry of Health & Welfare, Republic of Korea (grant number : HI17C1844010019).
